# Case Report: Motivational ambivalence and family dynamics in severe adolescent anorexia nervosa treated in a Mexican multidisciplinary setting

**DOI:** 10.3389/fpsyg.2026.1890098

**Published:** 2026-08-28

**Authors:** Jorge Armando Barriguete Meléndez, Mariana Valdez-Aguilar, Ana Regina Pérez Bustinzar, Cristina González Garcia, Paola Barriguete Chávez-Peón, Adriana Viladoms Portugal, Carmen Elisa Montes de Oca Moncada, Nancy Jiménez Miranda

**Affiliations:** 1 Instituto Nacional de Ciencias Medicas y Nutricion Salvador Zubiran, Mexico City, Mexico; 2 Department of Nutrition, Universidad Anáhuac, Mexico City, Mexico; 3 Clínica Ángeles TCA, Hospital Ángeles Lomas, Mexico City, Mexico; 4 Department of Psychology, Universidad Iberoamericana, Mexico City, Mexico

**Keywords:** adolescence, anorexia nervosa, case report, family intervention, motivation to change, multidisciplinary treatment

## Abstract

This case report describes the clinical course of two adolescent females (ages 12 and 14) with severe restrictive-type anorexia nervosa (AN) and comorbid anxiety and depressive symptoms. Both presented with extreme malnutrition (BMI-for-age <1st percentile), amenorrhea, distorted body image, and marked ambivalence toward weight restoration. Their families, characterized as structured, were engaged with the treatment. Care involved short-term hospitalization followed by intensive outpatient multidisciplinary treatment integrating cognitive-behavioral and dialectical behavior therapy strategies, nutritional rehabilitation, psychopharmacology, and structured caregiver involvement, alongside a psychodynamic-systemic family intervention informed by initial assessment. This model provided a framework to identify relational and emotional processes underlying motivational ambivalence. Over 6 months, both patients achieved weight restoration (approximately 50th–60th percentile BMI-for-age), with reduced eating disorder (ED) symptoms and improved emotional awareness, accompanied by changes in family functioning. However, residual anxiety, perfectionism, fluctuating insight, family conflict, and treatment fatigue persisted. One patient discontinued treatment prematurely despite clinical stabilization, with family and couple conflict. These cases highlight the complicated interaction between symptom remission, motivational ambivalence, and family dynamics in adolescent AN, emphasizing that weight restoration represent positive response, but alone does not constitute full recovery multidisciplinary, and family-focused interventions are essential.

## Introduction

Anorexia nervosa (AN) is a severe and potentially life-threatening psychiatric disorder with peak onset during adolescence, a developmental period characterized by identity formation, increasing autonomy, and heightened sensitivity to social evaluation (American Psychiatric Association [APA], 2013). In restrictive-type AN, starvation behaviors are frequently experienced as ego-syntonic, perceived as consistent with personal values such as discipline and control (Gregertsen et al., 2017). This ego-syntonic quality contributes to motivational ambivalence, as adolescents may simultaneously desire recovery and fear losing the perceived benefits of restriction (Budia et al., 2023; Robinson et al., 2024).

Current evidence-based guidelines, including the American Psychiatric Association (APA) Practice Guideline for the Treatment of Patients with Eating Disorders (ED) and the National Institute for Health and Care Excellence (NICE Guideline NG69), recommend multidisciplinary treatment integrating medical stabilization, nutritional rehabilitation, family involvement, and evidence-based psychotherapy for adolescents with AN. These recommendations informed the clinical management described in this report. Although weight restoration is a central treatment goal, research consistently shows that normalization of anthropometric indicators (e.g., BMI-for-age percentiles) does not necessarily reflect full psychological recovery. Residual symptoms may persist after physiological stabilization and increase the risk of relapse (Boone et al., 2012, 2013; Dahlenburg et al., 2019; Sander et al., 2021).

Family dynamics are central to treatment and recovery in adolescent AN (Barriguete Meléndez et al., 2026; Costa and Melnik, 2016; Salinas et al., 1992; Webb et al., 2022). Family-based approaches emphasize caregiver involvement in nutritional rehabilitation, emotional regulation, and treatment adherence, but also introduce challenges such as parental coping differences, relational tensions, and high-performance expectations that interact with adolescents’ perfectionism and identity development (Alharbi et al., 2024; Cerniglia et al., 2017; Karlstad et al., 2022). Recent case-based evidence further highlights that successful multidisciplinary treatment requires addressing not only medical and nutritional rehabilitation but also the interpersonal, familial, and psychosocial processes that influence recovery and long-term treatment engagement (Kovács and Boros, 2025). Unlike conventional family-based approaches that primarily focus on supporting nutritional rehabilitation and reducing eating disorder behaviors, the psychodynamic-systemic family model systematically evaluates communication patterns, emotional expression, parental roles, authority, individuation processes, relational conflicts, and intergenerational meanings. These domains inform individualized family interventions aimed at addressing relational processes that may contribute to motivational ambivalence, treatment adherence, and long-term recovery (Salinas et al., 1992; Pérez Bustinzar et al., 2024).

Despite extensive research on family-based and multidisciplinary treatments in high-income Western countries (Brewerton et al., 2020; Gorrell et al., 2019; Barriguete Meléndez et al., 2020), evidence from Mexico and broader Latin America remains limited (Barriguete-Meléndez et al., 2025; Dunker et al., 2023; Trujillo-Chivacuan and Perez, 2024), with studies highlighting high psychiatric comorbidity and complex clinical presentations (Ortega Luyando et al., 2025; Valdez-Aguilar et al., 2023).

This case report describes two adolescents with severe restrictive-type AN and highlights a divergence between symptomatic improvement and internalized motivational recovery within a Mexican multidisciplinary context. It is distinguished using a psychodynamic-systemic family model to examine how relational processes influence treatment adherence beyond weight restoration. By comparing two clinically similar cases with different outcomes, it contributes to understanding the role of family dynamics.

## Reporting standards

This case report was prepared in accordance with the CARE (CAse REport) Guidelines (Riley et al., 2017) to ensure comprehensive and transparent reporting of patient information, clinical findings, diagnostic assessment, therapeutic interventions, follow-up, and outcomes. The multidisciplinary treatment approach described in this report was consistent with internationally recognized recommendations for the assessment and treatment of adolescents with AN, including the American Psychiatric Association (APA) Practice Guideline for the Treatment of Patients with ED and the National Institute for Health and Care Excellence (NICE) Guideline NG69: Eating Disorders.

## Ethical considerations

Both patients received multidisciplinary clinical care as part of routine treatment at a specialized ED clinic. Written informed consent for publication of the clinical information was obtained from the legal guardians of both patients, and assent was obtained from the adolescents when appropriate for their age and developmental level. All potentially identifying information has been removed or anonymized to protect patient confidentiality. The publication of these cases was approved by the Human Ethics Committee of the ED Clinic and was conducted in accordance with the ethical principles of the Declaration of Helsinki and its subsequent amendments.

## Patient information

### Case 1

A 12-year-old female, only child, was referred after 7 months of progressive dietary restriction and rapid weight loss, triggered by persistent body-related teasing during her first year of junior high school. Prior to illness, she weighed approximately 68 kg and had recently experienced menarche at age 11; she subsequently lost nearly 30 kg, reaching a minimum weight of 39.7 kg. At intake, she reported an intense fear of “losing control” of her body and described pervasive preoccupation with food, calories, and body shape.

Her psychosocial context included high parental expectations regarding academic and social performance, along with multiple established risk factors for ED, including female sex, adolescence, family history of EDs, and a sociocultural environment that overvalued thinness.

Family assessment was conducted during hospitalization using a psychodynamic-systemic model (Salinas et al., 1992). Key areas identified for intervention included restricted communication patterns, limited expression of both positive affect and distress, elevated performance expectations within the family system, and overprotection of the patient. These domains informed a systemic formulation in which restrictive symptoms were understood as embedded within family communication patterns, affect regulation, and performance expectations.

### Case 2

A 14-year-old female was evaluated during hospitalization following admission to the intensive care unit for bradycardia secondary to severe malnutrition. Eight months prior, she had progressively reduced food intake while intensifying physical training. At presentation, she weighed 38 kg at a height of 1.66 m (BMI-for-age <1st percentile), reported consuming approximately 43% of estimated caloric requirements, and described fasting episodes of up to 48 h. Amenorrhea had been present for 6 months.

She was the eldest daughter in a two-parent household. Both parents expressed significant concern and actively sought care; although initially aligned around treatment, subtle differences in parental coping styles emerged early in the process. Her psychosocial context included exposure to a high-performance athletic environment emphasizing achievement without adequate nutritional or psychological support, a demanding academic setting, and experiences of peer bullying.

Family assessment was conducted during hospitalization using a psychodynamic-systemic model (Salinas et al., 1992). Key areas for intervention included a stalled individuation process within the nuclear family, communication difficulties between parents, family rigidity, and limited emotional expression across the system. These findings supported a systemic conceptualization in which symptom maintenance was linked to difficulties in individuation, emotional expression, and unresolved parental dynamics. Clinical summary of both cases is described in Table 1.

**Table 1:** Clinical summary. Baseline characteristics of the two patients.

Characteristics	Case 1	Case 2
Age	12 years	14 years
Sex	Female	Female
Primary diagnosis	DSM-5 anorexia nervosa, restrictive type	DSM-5 anorexia nervosa, restrictive type
Duration of illness before presentation	7 months	8 months
Reason for referral	Progressive weight loss and dietary restriction	Severe malnutrition with bradycardia requiring ICU admission
Lowest recorded weight	39.7 kg	38 kg
BMI-for-age	<1st percentile	<1st percentile
Menstrual status	Secondary amenorrhea	Secondary amenorrhea (6 months)
Medical complications	Severe malnutrition	Bradycardia, severe malnutrition
Previous treatment	None	Emergency medical stabilization

## Therapeutic intervention and clinical course

Treatment was delivered by a multidisciplinary team specialized in ED, including psychiatry, pediatrics, nutrition, psychology, and family therapy. The primary goals of treatment were medical stabilization, nutritional rehabilitation, restoration of healthy eating patterns, prevention of medical complications, and psychological recovery through coordinated multidisciplinary care.

Individual psychological treatment integrated cognitive behavioral therapy (CBT) and dialectical behavior therapy (DBT) techniques. CBT interventions focused on psychoeducation, self-monitoring of eating behaviors, cognitive restructuring of dysfunctional beliefs related to weight, body image, perfectionism, and control, and graduated exposure to feared foods. DBT techniques targeted mindfulness, emotion identification, distress tolerance, and emotion regulation skills to reduce anxiety associated with nutritional rehabilitation and improve coping with emotional distress.

Family intervention was conducted separately using the Psychodynamic-Systemic Family Assessment Model (Salinas et al., 1992). Unlike conventional family-based approaches primarily focused on supporting nutritional rehabilitation and symptom management, this model systematically evaluates communication patterns, emotional expression, parental roles, authority, individuation processes, relational conflicts, and intergenerational meanings. These domains were used to guide individualized family interventions aimed at addressing relational processes contributing to motivational ambivalence and treatment adherence.

### Case 1

The patient initially required a two-week hospitalization for medical and nutritional stabilization due to the severity of her weight loss and associated psychiatric symptoms. Following discharge, she entered an intensive outpatient multidisciplinary treatment program involving psychiatry, nutrition, psychology, and family therapy.

Psychotherapeutic treatment consisted of 27 weekly sessions using an integrative approach combining CBT and DBT. Early sessions prioritized the development of a strong therapeutic alliance through empathic validation and the creation of a safe environment for emotional expression.

Family intervention, bi-weekly family sessions, followed a psychodynamic-systemic approach focused on modifying communicational, authority and relational patterns. Interventions targeted emotional expression, intergenerational meanings around body and performance, and the differentiation of the adolescent’s identity from both the disorder and family expectations.

Throughout the early phase of treatment, the patient displayed high levels of anxiety related to food intake, body image, and physiological sensations associated with refeeding. Family sessions revealed that body-related concerns and weight-focused comments had also been present in the mother’s family of origin, which contributed to broader discussions about sociocultural and intergenerational influences on body image within the family context. Cognitively, the patient and family, demonstrated rigid beliefs linking thinness with personal worth, social and familiar acceptance. Common thoughts included fears of losing value if weight increased and beliefs that others expected her to remain thin.

During the first 3 months, alongside nutritional rehabilitation, interventions targeted physiological and emotional regulation using diaphragmatic breathing, mindfulness, and DBT-based distress tolerance. Pharmacological treatment was managed by the treating psychiatrist and included sertraline and clonazepam, with dosages adjusted according to clinical response and tolerability.

Cognitive restructuring was gradually introduced to address maladaptive beliefs about body image, self-worth, and control. By the third month of treatment, significant improvement in psychological symptoms was observed. The patient showed increased emotional awareness and greater capacity to identify disordered thoughts.

Despite these improvements, residual safety behaviors persisted, including body checking, social comparison, and reassurance seeking. Treatment therefore shifted toward reducing these behaviors while fostering self-compassion, cognitive flexibility, and broader identity development. Family sessions remained central, supporting a transition from monitoring to a more collaborative and supportive caregiving approach. Over time, monthly sessions were associated with improved emotional expression and greater openness to discussing distress and disagreements. Parents also developed increased awareness of the impact of family comments and worked with the patient to manage them more effectively.

### Case 2

The patient’s treatment began in an intensive care setting due to severe medical instability associated with malnutrition and bradycardia. Initial management focused on medical stabilization and nutritional rehabilitation, and psychiatric medical treatment. During this phase, the patient required close monitoring and received both parenteral and enteral nutritional support. Pharmacological treatment was initiated to address depressive symptoms, anxiety, and cognitive rigidity and included fluoxetine, olanzapine, and clonazepam for anxiety and sleep disturbances. Medication dosages were adjusted throughout treatment according to clinical response and tolerability.

During hospitalization, which lasted approximately 2 weeks, treatment was delivered by a multidisciplinary team including pediatrics, psychiatry, nutrition, and psychology with a motivational and psychoeducational approach. A structured nutritional plan was implemented under continuous supervision, supported by a 24-h caregiver responsible for monitoring meals and medication adherence. Behavioral contracts were introduced to reinforce treatment adherence. Despite severe medical compromise, the patient initially demonstrated intense fear of food intake and significant distress associated with increasing meal portions, frequently reporting abdominal discomfort and anxiety during the refeeding process.

Following hospital discharge, the patient transitioned to intensive outpatient care with continued 24-h caregiver supervision at home. Early outpatient treatment was characterized by frustration and resistance, particularly regarding restrictions on physical activity, delayed return to competitive swimming, and limitations on school and social activities. These constraints contributed to low intrinsic motivation for change and emotional tension within the family environment.

Over the first 3 months of treatment, gradual nutritional rehabilitation and weight restoration were achieved. Anthropometric indicators improved progressively, reaching approximately the 50th percentile for BMI-for-age, indicating recovery from the acute risk of malnutrition. Concurrently, laboratory parameters normalized and standardized psychological measures showed substantial reductions in ED symptomatology.

Personal psychological interventions during this phase increasingly focused on emotional identification, distress tolerance, and cognitive restructuring of beliefs related to control, body image, and performance. Family involvement was central. Both parents participated actively in psychoeducation and family sessions and initially appeared aligned in prioritizing recovery. As the acute medical phase stabilized, differences in coping styles became more evident: the mother reverted to previous parenting behaviors characterized by a controlling and rigid approach to monitoring routines, whereas the father demonstrated a more avoidant parenting style, at times compromising consistency.

By 6 months of treatment, the patient maintained anthropometric parameters within the healthy range for age, with body composition indicators also falling within expected limits. Standardized symptom measures remained below clinical thresholds and overt restrictive behaviors had ceased. Improvements were also observed in emotional awareness and expression.

As symptoms improved, family work revealed broader systemic difficulties, including limited emotional expression—both positive and conflict-related—and longstanding marital conflict characterized by poor communication, unresolved tensions, and underlying anger. Interventions increasingly focused on the parental relationship; however, as these issues became more salient, the parents chose to discontinue family work.

## Diagnostic assessments

Both adolescents underwent a comprehensive multidisciplinary assessment at admission, including medical, psychiatric, nutritional, psychological, and family evaluations. The diagnostic process included a comprehensive clinical diagnostic interview conducted by a board-certified psychiatrist, together with a detailed psychiatric history, mental status examination, and collateral information obtained from the patients and their caregivers. Psychiatric diagnoses were established according to the DSM-5 criteria for AN. Medical evaluation included physical examination, anthropometric measurements, laboratory testing, and cardiac assessment to determine medical stability and the need for hospitalization.

As part of the initial multidisciplinary assessment, family functioning was systematically evaluated using the Psychodynamic-Systemic Family Assessment Model (Salinas et al., 1992). This assessment explored family structure, communication patterns, emotional expression, parental roles, authority, individuation processes, relational conflicts, and intergenerational dynamics.

Psychological assessment was conducted by licensed clinical psychologists specialized in ED using a standardized assessment battery. The battery included the Eating Attitudes Test (EAT), the Eating Disorder Inventory-3 (EDI-3), Bulimia Test (BULIT), Hamilton Depression Scale (HDA), and the Stages of Change Questionnaire (ACTA) to assess eating disorder symptomatology, depressive symptoms, and readiness for change. Comprehensive multidisciplinary reassessments were conducted at 3 and 6 months after treatment initiation. These follow-up evaluations integrated medical, nutritional, psychiatric, psychological, and family assessments to monitor treatment response, including weight restoration, eating behaviors, emotional functioning, treatment adherence, and family dynamics. Clinical improvement was determined through the integration of findings from these multidisciplinary evaluations.

## Follow-up and outcomes

### Case 1

Following the intensive six-month phase, care shifted to maintenance. Weight remained stable within the healthy range, with no recurrence of restriction, food avoidance, or compensatory behaviors. As caregiver supervision was gradually reduced, the patient maintained nutritional adherence independently, allowing psychotherapy to decrease from weekly to biweekly sessions.

During periods of academic stress, mild increases in anxiety and perfectionism were observed, without behavioral relapse. At last follow-up, anthropometric parameters remained stable and menstrual function had resumed and remained regular, with no adverse medical events. Family treatment was associated with improved communication and emotional expression, with greater capacity to share and validate feelings.

The family also shifted away from appearance-focused comments toward valuing abilities and personal qualities. Expectations decreased, overprotection diminished, and the patient achieved greater autonomy and independence across domains.

### Case 2

In the months following weight normalization, anthropometric and laboratory values remained stable. Cholesterol levels and endocrine markers improved progressively. Menstrual function resumed once during follow-up. Pharmacological treatment was maintained during this phase with good tolerability and no significant adverse effects. Nutritional adherence remained consistent after reduction of caregiver supervision, and no restrictive relapse was documented. However, as treatment intensity decreased, family relational stress resurfaced.

Despite maintained weight and normalized symptom scores, emotional disengagement from psychotherapy increased. Ultimately, treatment was discontinued. At the time of last contact, weight and laboratory values remained within normal limits. No acute medical complications were present. Long-term outcomes beyond 6 months are unknown. A comparative summary of clinical progression, interventions, and family dynamics across both cases is presented in Table 2.

**Table 2:** Clinical timeline across Case 1 and Case 2.

Timepoint	Case 1–Clinical/interventions family/outcomes	Case 2–Clinical/interventions/family/outcomes
Baseline (Admission)	Severe malnutrition (BMI < 1st percentile), amenorrhea, anxiety. Hospitalization + refeeding. Family structured, limited emotional expression. → Medical stabilization initiated	Severe malnutrition, ICU admission, bradycardia. Hospitalization + pharmacotherapy. Parents initially aligned. → Medical stabilization initiated
0–3 months	Weight gain with distress during refeeding. CBT/DBT + emotional regulation. Family work begins. → Reduction in ED behaviors	Weight restoration begins under supervision. Intensive outpatient care. Emerging parental coping differences. → Symptom reduction
3–6 months	Weight normalized, ↓ ED symptoms. Cognitive restructuring + identity work. Improved communication, ↓ overprotection. → Psychological improvement	Weight normalized, ↓ ED symptoms. Cognitive + emotional work. Marital conflict emerges. → Persistent ambivalence
6 months / End phase	Stable weight, improved emotional awareness. Reduced therapy intensity. Family cohesion ↑. → Continued recovery	Stable weight, normalized labs. Reduced supervision. Family conflict intensifies. → Emotional disengagement
Follow-up	Stable weight, no relapse, menstrual recovery maintained. Family supports autonomy and validation. → Sustained recovery	Stable weight, no medical relapse. Treatment discontinued. Family conflict unresolved. → Dropout despite stabilization

## Patient perspective

Patients and families described the early stages of treatment, particularly nutritional rehabilitation and refeeding, as emotionally challenging. Both adolescents reported that, over time, treatment helped them gradually re-establish regular eating patterns, better understand the relationship between emotions and eating behaviors, and increase their willingness to engage in recovery, although fear of weight gain and ambivalence toward recovery persisted to varying degrees. Families perceived improvements in communication, emotional understanding, and parental support throughout treatment. In Case 1, these changes were associated with sustained engagement in treatment and greater autonomy. In Case 2, despite physical stabilization and reduced restrictive behaviors, persistent family conflict and emotional disengagement contributed to treatment discontinuation.

## Discussion

This case report illustrates a divergence between symptomatic improvement and internalized motivational recovery in adolescents with severe restrictive-type AN within a Mexican multidisciplinary context. Although weight restoration and symptom reduction were achieved, motivational ambivalence persisted, with family dynamics playing a key role in treatment adherence and continuity.

### Weight restoration and psychological recovery

Both adolescents achieved weight normalization within 6 months, consistent with evidence demonstrating the effectiveness of family-based and multidisciplinary interventions in promoting short-term weight gain in adolescent AN (Cerniglia et al., 2017; Lock and Le Grange, 2019; Solmi et al., 2024). However, in line with longitudinal research, physiological recovery in our cases did not fully coincide with cognitive remission. Bardone-Cone et al. (2010) emphasized that full recovery requires normalization not only of weight but also of eating disorder cognitions and behaviors (Bardone-Cone et al., 2010). Similarly, one study reported that individuals meeting weight criteria may continue to experience residual body dissatisfaction and fear of weight gain, factors associated with relapse (Keel et al., 2007).

Furthermore different studies had documented elevated relapse rates within the first year following weight restoration, particularly when cognitive symptoms persist (de Rijk et al., 2024; Heal-Cohen et al., 2025). Consistent with these findings, both adolescents in our report demonstrated normalized anthropometric indicators and reduced symptom scores, yet perfectionistic thinking and conditional self-worth remained evident. These findings suggest that behavioral and physiological recovery may occur in the absence of deeper relational and emotional change, which are not directly targeted by symptom-focused interventions.

### Motivational ambivalence and identity processes

Despite behavioral compliance under supervision, both adolescents exhibited persistent fear of weight gain and attachment to restrictive identity. This pattern aligns with conceptualizations of AN as an ego-syntonic disorder, in which restrictive behaviors are perceived as congruent with valued aspects of identity such as discipline and control (Gregertsen et al., 2017), Recent systematic reviews highlight substantial individual variability in motivation to change among individuals with EDs, with ambivalence frequently predicting poorer treatment engagement (Hoetzel et al., 2013; Robinson et al., 2024; St-Hilaire et al., 2017). In our cases, adherence during early phases appeared externally regulated, and deeper motivational integration required sustained therapeutic engagement. This distinction between behavioral remission and autonomous motivation may partially explain the treatment discontinuation observed in Case 2 despite medical stabilization.

### Family relational dynamics and treatment continuity

Strong empirical evidence supports parental participation as a cornerstone of adolescent AN treatment (Beintner et al., 2020; Karlstad et al., 2022; Lock and Le Grange, 2019). In both cases, structured caregiver supervision supported early nutritional stabilization and the interruption of restrictive behaviors.

However, family processes may also influence variability in treatment engagement and long-term outcomes. Caregiver emotion regulation and parental alignment have been shown to affect adherence to treatment, while high expressed emotion and unresolved relational conflict are associated with increased dropout and relapse risk (Waage et al., 2025). In Case 2, the emergence of parental tension during therapy coincided with progressive disengagement from treatment despite maintained medical stability. This observation suggests that, beyond behavioral refeeding, sustained recovery may require addressing broader relational dynamics within the family system. The psychodynamic-systemic model allowed for the identification of these underlying relational processes, highlighting that family involvement is not inherently protective, but contingent on the system’s capacity for emotional and structural change.

**Figure 1:**
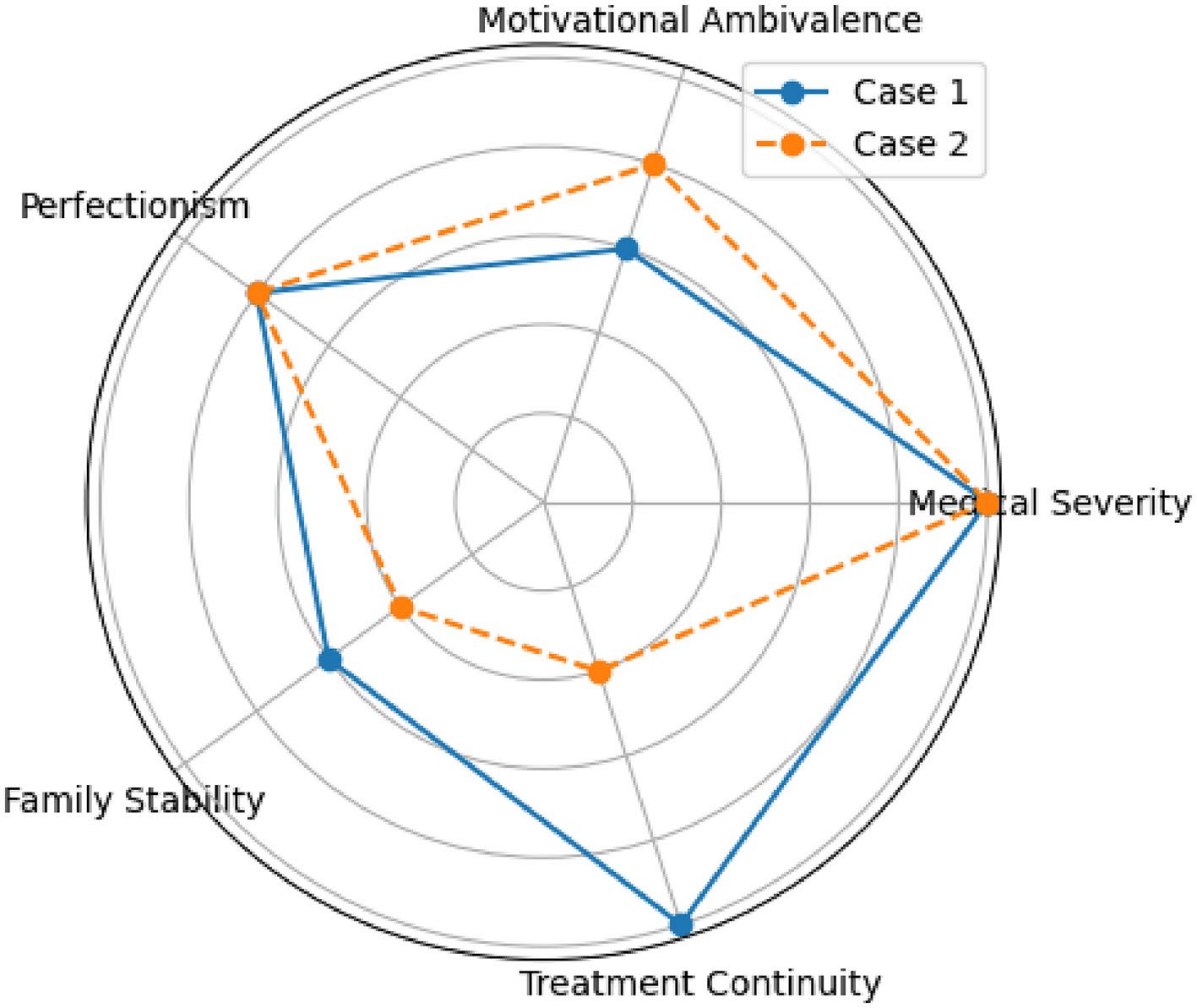
Conceptual clinical formulation derived from comparative case analysis. Domains were qualitatively rated (0–5) based on structured clinical assessment across treatment phases and are presented for illustrative purposes only. The figure represents a conceptual synthesis.

To synthesize these interacting domains, Figure 1 presents a conceptual clinical formulation derived from comparative case analysis.

### Clinical implications

These findings underscore that, in adolescent AN, early weight restoration—while essential—does not ensure motivational or relational recovery. Durable remission requires addressing identity processes, perfectionism, emotion regulation, and family system dynamics, particularly in high-performance contexts.

Importantly, this report highlights the added value of a psychodynamic-systemic family evaluation and treatment model, which extends beyond behavioral management by targeting emotional communication, individuation processes, and underlying relational conflicts. Unlike more symptom-focused approaches, this model allows for the identification of deeper systemic barriers to recovery, including rigid family structures and unresolved parental dynamics that may sustain motivational ambivalence, as well as the belief that weight restoration alone is sufficient treatment.

Family interventions guided by this model were associated with not only in the patient but across the family system. However, a key observation was variability in family readiness for systemic change: while some families engaged in broader relational transformation, others restricted their involvement to behavioral and nutritional aspects of care. This suggests that the effectiveness of family-based interventions are important, but are moderated by the family’s structural flexibility and capacity to tolerate emotional change.

This case report has several strengths. It provides a detailed description of two adolescents with severe AN treated within a real-world multidisciplinary setting, illustrating how medical, nutritional, psychiatric, psychological, and family interventions were integrated throughout treatment. In addition, the report offers a detailed application of the psychodynamic-systemic family assessment model, highlighting how family relational processes informed individualized treatment planning in routine clinical practice. By presenting two contrasting clinical trajectories, the report also contributes to understanding the complexity of motivational ambivalence and family dynamics in adolescent AN.

Nevertheless, several limitations should be acknowledged. First, the findings are based on only two clinical cases, limiting the generalizability of the observations. Second, although family dynamics were systematically explored through the psychodynamic-systemic family assessment, standardized measures of family functioning were not administered, limiting the objective evaluation of changes in family processes over time. Third, follow-up was limited to the treatment period reported in this manuscript, preventing conclusions regarding the long-term maintenance of treatment outcomes. In addition, the psychodynamic-systemic formulation represents a clinical interpretative framework and should therefore be considered descriptive rather than explanatory. Consequently, causal relationships between family dynamics, motivational ambivalence, and treatment outcomes cannot be established. Future studies including larger samples, standardized assessments of family functioning, and longer longitudinal follow-up are needed to further evaluate these relationships.

## Conclusion

This case report highlights the importance of a multidisciplinary approach to adolescent anorexia nervosa and underscores the potential value of incorporating psychodynamic-systemic family assessment to better understand motivational ambivalence and family relational processes. Further research with larger samples and longitudinal designs is needed to evaluate its clinical contribution.

## Data Availability

The original contributions presented in the study are included in the article/supplementary material, further inquiries can be directed to the corresponding author.
